# Multidimensional Evaluation of Myofascial Pelvic Pain and Other Comorbidities in Endometriosis Patients

**DOI:** 10.3390/jcm14103455

**Published:** 2025-05-15

**Authors:** Lara Quintas-Marquès, Marta Valdés-Bango, Carla Box, Meritxell Gràcia, Mariona Rius, Francisco Carmona, Maria-Ángeles Martínez-Zamora

**Affiliations:** Gynecology Department, Institute Clinic of Gynecology, Obstetrics and Neonatology, Hospital Clinic, Universitat de Barcelona, Institut d’Investigacions Biomèdiques August Pi i Sunyer (IDIBAPS), 08036 Barcelona, Spain; lquintas@clinic.cat (L.Q.-M.); cebox@clinic.cat (C.B.); megracia@clinic.cat (M.G.); marius@clinic.cat (M.R.); fcarmona@clinic.cat (F.C.)

**Keywords:** myofascial pelvic pain, endometriosis, pain comorbidities, endometriosis-associated pain

## Abstract

**Background/Objectives**: Pain associated with endometriosis is complex and influenced by multiple factors. The presence of myofascial pelvic pain (MPP), associated comorbidities, and overlapping symptoms may play a role in endometriosis-associated pain. The aim of this study was to evaluate MPP in patients with endometriosis and correlate it with other self-reported comorbidities and symptoms, health-related quality of life, and mental health. **Methods**: A cross-sectional study was carried out at a tertiary hospital referral center on 175 women with endometriosis. MPP was evaluated by clinical examination; if present, the patient was allocated to the MPP group (*n* = 84), and if absent, the patient was allocated to the non-MPP group (*n* = 91). Other comorbidities and symptoms frequently found in patients with chronic pain were also recorded. The Short Form 36-Item health questionnaire (SF-36) and the Hospital Anxiety and Depression Scale (HADS) were administered. Central sensitization-related symptoms were assessed using the Central Sensitization Inventory (CSI). **Results**: Patients with MPP showed significantly higher scores related to endometriosis-associated pain, reported lower scores in all domains of the SF-36, and higher scores in the HADS questionnaire. This group also reported more comorbid symptoms and showed higher scores in the CSI questionnaire. In the multivariate analysis, severe non-cyclic pelvic pain, abdominal bloating, and CSI ≥ 40 remained significantly associated with the presence of MPP. **Conclusions**: Endometriosis patients with MPP presented more pain comorbidities and general symptoms. Moreover, they reported more endometriosis-associated pain and worse health-related quality of life, and they may be at higher risk of depression and anxiety.

## 1. Introduction

Endometriosis is a leading cause of pain among women and can severely affect health-related quality of life (HRQoL) [1]. The association between endometriosis and pelvic pain is widely known, but the specific mechanisms by which endometriosis causes pain are still poorly understood [2]. Furthermore, in recent years, it has been described that endometriosis patients frequently present pain across various sites or are diagnosed with concurrent pain conditions. Nearly 95% of women with endometriosis report at least one or more comorbid disorders, such as migraine, depression, anxiety, irritable bowel syndrome, myofascial pain, or fibromyalgia [3]. All these comorbid pain conditions may play a role in poor or partial medical and/or surgical outcomes, and there are reports of low HRQoL by many of these patients.

Myofascial pain is a regional musculoskeletal condition arising from hyperirritable spots or contraction knots in skeletal muscles or their fascia, termed myofascial trigger points (TrPs) [4]. Myofascial pain is frequently present in patients presenting chronic pain conditions, such as bladder pain syndrome or fibromyalgia [5,6]. Moreover, it has been shown that myofascial pain can impact treatment outcomes and the quality of life of these patients [7].

Myofascial TrPs can occur secondary to muscle overload (primary dysfunction), but they may also be present as a secondary phenomenon associated with painful underlying visceral events; for example, TrPs may develop secondary to a visceral disease, such as endometriosis [8,9]. In this case, TrPs occur in muscle structures located in the referred pain area from a specific viscera, especially when the visceral allogenic process has been particularly intense or repetitive. The diagnosis of myofascial pelvic pain (MPP) relies entirely on clinical criteria, involving both a review of the medical history and a physical examination of the affected muscles [10]. Myofascial pain has traditionally been categorized as nociceptive pain; however, it has recently been suggested that this condition could be present in patients with predominantly nociplastic pain [11] and that central sensitization (CS) may contribute to its pathogenesis [12,13]. On the other hand, it is known that chronic pain is connected with psychological factors and psychiatric comorbidities [14]. Co-occurring symptoms of CS, including fatigue, disturbed sleep [15], and cognitive problems, are also frequently reported among patients with chronic pelvic pain. Moreover, the existence of several comorbidities not only increases the risk of developing other chronic conditions but has also been demonstrated to play a role in the prolonged presence of pain and unsuccessful treatment outcomes [16].

Despite its high prevalence among individuals with chronic pelvic pain, MPP remains one of the most frequently overlooked diagnoses in patients with endometriosis, and it is not systematically assessed in these patients. Previous, albeit scarce, research suggested that endometriosis-associated chronic pelvic pain may frequently be related to the presence of MPP [8,17]. Research evaluating MPP syndrome in endometriosis patients is needed to gain a deeper understanding of the development and maintenance of chronic pelvic pain in these patients, as well as for devising effective management strategies for this syndrome.

The objective of this study was to evaluate the clinical and demographic characteristics of patients with endometriosis and MPP syndrome and also analyze whether the presence of MPP in endometriosis patients influences other symptoms and comorbidities, HRQoL, and mental health.

## 2. Materials and Methods

### 2.1. Study Design

This observational cross-sectional study was conducted at the Hospital Clinic of Barcelona, a tertiary hospital serving as a national center for endometriosis. Data were collected from September 2023 to March 2024. The study was approved by the Clinical Research Ethics Committee of the Hospital Clinic of Barcelona (reference HCB 2021/0674). All women gave written informed consent before the initiation of the study.

### 2.2. Patients

All consecutive new patients aged 18 years or older with suspected endometriosis, referred to the Department of Gynecology for treatment, underwent a clinical examination and two–three-dimensional transvaginal ultrasound (TVUS) between September 2023 and March 2024. Those diagnosed with endometriosis were included in the study. MPP was evaluated; if present, the patient was allocated to the MPP group, and if absent, they were allocated to the non-MPP group. Exclusion criteria were malignancy or a history of malignancy, endocrine, cardiovascular, autoimmune, or systemic inflammatory diseases; premature ovarian failure; menopausal status; or previous hysterectomy.

### 2.3. Study Procedures and Data Collection

All women underwent clinical examination and a TVUS using an endovaginal probe (type RIC5-9, Voluson S10; GE Healthcare, Milwaukee, WI, USA). The presence of ovarian and deep endometriosis was assessed according to the International Deep Endometriosis Analysis group’s consensus [18,19].

The following clinical and epidemiological data of each patient were collected: age, body mass index, tobacco consumption, and the use of medical treatments. The pain intensity experienced by patients was quantitatively assessed using the Numerical Rating Scale (NRS) from 0 (no pain) to 10 (unbearable pain). This scale allows patients to subjectively rate their pain, providing an effective measure of the symptom’s severity. Different types of pain were assessed: dysmenorrhea, dyspareunia, and non-cyclical chronic pelvic pain (NCPP). NCPP was defined as the presence of intermittent or permanent pelvic pain not related to the menstrual cycle for at least 6 months [20]. The score can either be used as a numerical variable or dichotomized as a categorical variable. We dichotomized the data, stratifying patients into groups with severe NCPP (NRS ≥ 7) and those with mild/moderate NCPP (NRS < 7).

All participants underwent clinical examination in the dorsal lithotomy position. Tenderness, spasms, and reproductions of pain symptoms were examined during the baseline gynecologist assessment via unidigital pelvic examination, as described by Meister et al. [21]. All muscle assessments were performed by the same gynecologist (L.Q-M). The examiner individually palpated the pelvic floor muscles intravaginally, starting with the deep muscular plane (palpation of the internal obturator muscle bilaterally at 11 and 1 o’clock) and moving superficially with the superficial perineal muscles (palpation of the levator ani bilaterally at 3 and 9 o’clock). A single digit was used to palpate each muscle once in the center of the muscle belly and then sweeping along the length of the muscle in the direction of the muscle fibers. The patient was asked if the palpation of different muscles provoked pain (yes/no) and if the pain produced by the pressure was mild (NRS 1–3), moderate (4–6), or severe (NRS 7–10); then, they were asked if the actions reproduced their main pain complaint. MPP was diagnosed as tenderness and a reproduction of moderate to severe pain symptoms in at least 2 of 4 pelvic floor muscles (right or left levator ani muscle complex and obturator internus muscle) [21].

Other comorbidities and common symptoms frequently found in patients with chronic pain were also recorded, such as headache, fatigue, and insomnia; arthralgia; and generalized muscle pain, as well as other common gastrointestinal symptoms.

All patients recruited completed the Spanish validated versions of three questionnaires: the Short Form 36-item (SF-36) [22], the Hospital Anxiety and Depression Scale (HADS) [23], and the Central Sensitization Inventory (CSI) [24].

The SF-36 questionnaire is a self-administered instrument for evaluating the general HRQoL [22]. The questionnaire comprises 36 items and assesses eight domains of health statuses: physical functioning, role limitations due to physical health, body pain, energy/fatigue, role limitations due to emotional problems, emotional well-being, social functioning, and general health perception.

The HADS questionnaire is a 14-item self-reported screening scale, with a 7-item anxiety subscale and a 7-item depression subscale. It has a four-point (0–3) response category, with a maximum score of 21 for depression and anxiety. Higher scores show poorer psychological status. Scores between 0 and 7 represent “no case”; 8 to 10 indicate “possible case”; 11 to 21 suggest “probable case”. Within this range, scores of 11–15 indicate moderate depression/anxiety, while scores of 16 or higher represent severe depression/anxiety; a score that is greater than or equal to 16 represents severe depression/anxiety [23].

CS-related symptoms were measured with the CSI questionnaire [24], which is a valid and reliable self-reported screening instrument developed to help identify patients presenting symptoms that may be related to CS [25,26]. It measures somatic and emotional symptoms related to CS. The CSI questionnaire assesses 25 symptoms, and each symptom is rated as never, rarely, sometimes, often, or always (0–4). Scoring ranges from 0 to 100, with a CSI cutoff point of ≥40 being considered the threshold of clinical relevance [26]. It has been used previously in endometriosis patients [27,28].

### 2.4. Statistical Analysis

The sample size was arbitrarily decided, albeit in keeping with previous studies analyzing MPP in patients with chronic pelvic pain. A minimum of 80 patients per group was proposed [14]. Statistical analysis was performed with the Statistical Package for the Social Sciences software, release 27.0, for Windows (SPSS, Chicago, IL, USA).

Continuous variables were expressed as mean ± standard deviation, and categorical variables were expressed as a number (percentage). Student’s *t*-test was used for comparisons of normally distributed continuous variables. The chi-square test was used for the comparison of categorical variables. Statistical significance was set at *p* < 0.05. Statistically significant differences among epidemiologic variables, clinical characteristics, or comorbid symptoms were evaluated as independent risk factors using multivariate analysis with logistic regression. Odds ratios and their 95% confidence intervals were reported.

## 3. Results

A total of 200 consecutive women with suspected endometriosis were invited to participate in the study. Among these patients, 175 were finally diagnosed with endometriosis by the TVUS exam, and all were accepted for participation in this study. Eighty-four patients were allocated to the MPP group, and 91 were allocated to the non-MPP group.

The clinical and epidemiological characteristics of the women studied are shown in Table 1. The women in the MPP group reported more gynecological pain symptoms than those in the non-MPP group (Table 1). In the MPP group, 42.8% of patients reported severe NCPP. There were no differences in the epidemiological characteristics, the type of endometriosis, or the number of previous endometriosis surgical interventions between the two groups. There were no differences between the two groups in relation to hormonal treatment. Endometriosis patients with MPP reported a higher use of opioid treatment (*p* = 0.007) (Table 1).

The comorbidities and other overlapping symptoms evaluated in both groups are described in Table 2. The MPP group showed a higher presence of other chronic pain comorbidities, such as fatigue and insomnia (60.9% vs. 43.8%; *p* = 0.032), as well as more generalized muscle pain (61.7% vs. 38%; *p* = 0.003) and osteoarticular pain (41.7% vs. 19%; *p* = 0.002). Headaches were also more present in the MPP group (64% vs. 44%; *p* = 0.014), and gastrointestinal symptoms, such as intestinal rhythm alterations, nausea/vomiting, or abdominal bloating, were also more frequently described in this group.

There were statistically significant differences between the two groups in HRQoL (Table 3). The MPP group presented lower scores in all dimensions of the SF-36 questionnaire, and the mean HADS scores were higher in both subscales in this group.

CSI scores were significantly higher in the MPP group (50.8 points vs. 37.3 points; *p* < 0.001). The MPP group presented a higher number of patients (79.7%; *p* < 0.001) with a CSI score above the diagnostic cutoff (CSI ≥ 40 points).

In the multivariate analysis, severe NCPP, abdominal bloating, and CSI of ≥40 remained significantly associated with the presence of MPP (Table 4).

## 4. Discussion

This study explored the presence of MPP among patients with endometriosis and its correlation with socio-demographic parameters; clinical symptoms; self-reported comorbid symptoms; other chronic pain conditions; HRQoL; and mental health.

The main findings showed that endometriosis patients presenting MPP have a higher prevalence of pain-related comorbidities and associated symptoms. These patients reported more severe endometriosis-related pain, with greater dysmenorrhea, NCPP, and dyspareunia, and a poorer HRQoL. Additionally, these women are at a significantly higher risk of developing depression and anxiety.

The diagnosis of MPP has been described in patients with endometriosis, and it has also been suggested that MPP may be related to CS [8]. The presence of persistent pain, including visceral or myofascial sources, can lead to the increased sensitization of the central nervous system and predispose these patients to the development of additional chronic pain conditions [29,30]. The presence of myofascial TrPs can act as a pain generator and continue to transmit sensory information to the central nervous system, even after the initial peripheral pathology has been resolved [17]. Myofascial pain often acts as a primary source of pain, but increasing evidence suggests that it can also coexist with various pathologies, thereby adding to the symptomatic burden [31]. When myofascial pain is diagnosed in patients who present another condition, we consider myofascial pain as a comorbid condition. In these situations, myofascial pain should be considered a comorbid condition that can contribute to the patient’s symptoms, rather than being the primary cause of the diagnosis. Our data are concordant with previous published research and suggest that the presence of MPP may not only be related to CS but also that MPP itself may be a cause of endometriosis-associated pain.

Our study found that endometriosis patients with MPP have a higher rate of opioid consumption, which can be explained by the increased pain they experience. MPP can act as an alert factor for identifying patients who may misuse opioids. Patients managing pain related to endometriosis through opioid use might constitute a subgroup with increased disease burden and comorbidities, warranting a more cautious therapeutic strategy [32].

Other comorbid symptoms frequently found in patients with chronic pain were also more frequent in the MPP group. Insomnia and sleep disturbances are commonly reported in patients with chronic pain. Previous research has shown that these patients experience sleep disturbances not only in terms of sleep onset or duration but also in experiencing non-restorative sleep, which is particularly relevant in chronic overlapping pain conditions and CS [33]. Fatigue is a frequently overlooked symptom in the management of patients with chronic pain, despite being as distressing as pain itself [34]. It is also believed to play a key role in the relationship between pain, psychosocial well-being, and HRQoL [35]. In our study, fatigue was more frequently reported among the MPP group.

The higher reported presence of generalized muscle pain, osteoarticular pain, and headaches in patients with MPP compared to the non-MPP group suggests a potential systemic involvement that goes beyond the pelvic region [36]. These symptoms may be indicative of a shared pathophysiological mechanism, in which chronic inflammatory processes and neural sensitization associated with endometriosis contribute to a heightened pain response in multiple body regions [37]. The interplay between endometriosis and MPP could lead to CS, in which pain perception is amplified due to persistent stimulation of the nervous system.

Patients with endometriosis frequently reported gastrointestinal symptoms such as abdominal bloating, nausea, and intestinal rhythm alterations (Table 2). These symptoms are notably more prevalent among individuals diagnosed with MPP, suggesting a complex interrelationship between gastrointestinal dysfunction and pelvic pain syndromes. The increased frequency of these symptoms in the MPP group suggests that potential mechanisms are in play, such as higher visceral sensitivity and neural cross-talk between pelvic and abdominal regions [38]. Understanding these interactions is critical for developing targeted interventions that address both the gynecological and gastrointestinal aspects of endometriosis.

The link between chronic pain and mental health conditions is well documented and is commonly considered to be bidirectional [39]. Our results revealed a significant psychosocial burden in patients with MPP, aligning with previous findings [14]. Furthermore, the higher prevalence of mental disorders and psychopathologies in these patients, along with the chronic pain they experience, may be related to a lower quality of life. Mental health conditions, such as anxiety and depression, appear to predispose patients to developing MPP [14]. This indicates that these conditions should be assessed and treated concomitantly in individuals with this symptom.

The multivariate analysis identified severe NCPP, abdominal bloating, and a CSI of ≥40 as significant predictors of MPP in patients with endometriosis. These findings highlight the complex interplay between MPP and overlapping conditions, suggesting a multidimensional pathophysiology that extends beyond endometriosis alone.

The above notwithstanding, the presence of MPP alongside other pathologies has significant implications for both diagnosis and treatment. Endometriosis is a very prevalent pathology, and therefore, apart from the adequate management of the pathology itself, it is important to establish tools for the early detection and specific treatment of MPP. Assessing MPP in endometriosis patients may improve the understanding of endometriosis-associated pain and facilitate a comprehensive evaluation of chronic pain.

Treatments centered on peripheral strategies aiming to treat MPP have demonstrated short-term effects but minimal long-term benefits. Therapies addressing the myofascial aspect of chronic pain should be contemplated as integral components within a multimodal program primarily targeting the underlying nociplastic pain mechanism [11]. The management of endometriosis patients with mixed nociceptive and nociplastic pain and other associated comorbid symptoms should be carried out with a multimodal approach, including, on the one hand, the management of peripheral nociceptive inputs (endometriosis lesions and TrPs), since removing the peripheral nociceptive input could potentially have the effect of modulating the central nervous pathways and, on the other hand, the management of central pain, including pain education and pharmaceutical or interventional treatments [40].

The present study has several strengths. First, to our knowledge, this is the first study to evaluate the presence of MPP in patients with endometriosis and correlate the findings with other comorbid symptoms, HRQoL, mental health, and CS-related symptoms. Second, the clinical examination for the assessment of MPP was performed by the same experienced physician following a standardized examination [21]. Third, all questionnaires used were standardized and validated in Spanish. The presence of endometriosis lesions was exhaustively evaluated in all patients, and the self-reported diagnosis of endometriosis was avoided.

This study also has several limitations. On the one hand, it included a group of patients with endometriosis from a tertiary referral center where more severe patients are evaluated. This could have led to an overestimation of the reported symptoms of MPP and other comorbid conditions in patients with endometriosis. Consequently, the findings of this study might be specifically relevant to comparable large referral centers with similar patient populations and demographics. On the other hand, due to the design of the study, the impact of medical or surgical treatment on MPP and other comorbid symptoms was not evaluated. Finally, as MPP remains a clinical diagnosis without currently available imaging or biomarker confirmation, our study shares this universal field limitation, compounded by the absence of standardized diagnostic criteria. To minimize inherent bias and ensure methodological rigor, all assessments were performed by the same pelvic pain specialist using a systematic evaluation approach derived from Meister et al. [21].

## 5. Conclusions

Endometriosis patients with MPP presented more pain comorbidities and general symptoms. Moreover, they reported more endometriosis-associated pain and worsened HRQoL, and they may be at higher risk of depression and anxiety.

Therefore, the evaluation of a patient diagnosed with endometriosis should include extensive assessments aimed at identifying all potential sources of pain, including MPP, psychological factors, and the presence of other comorbidities and other common symptoms. Identifying these patients and providing a multimodal pain-centered treatment approach, including physiotherapeutic and psychotherapeutic management, is recommended. Future studies should assess whether this multidimensional approach leads to improved long-term outcomes.

## Figures and Tables

**Table 1:** Clinical and epidemiological characteristics of the two groups analyzed.

	MPP Group (*n* = 84)	Non-MPP Group (*n* = 91)	*p*-Value
Age, years, mean (SD)	36.7 ± 6.7	37.69± 5.6	0.36
BMI, kg/m^2^, mean (SD)	24.7 ± 5.3	24.9 ± 4.4	0.776
Tobacco use (y/n)	13 (15.4%)	12 (13.1%)	0.673
Hormonal treatment	61 (72.6%)	60 (65.9%)	0.413
Opioid treatment	9 (10.7%)	1 (1.1%)	0.007
Previous endometriosis surgeries	44 (52%)	42 (46%)	0.451
Type of endometriosis			
Ovarian endometrioma	50 (59%)	58 (63.7%)	0.641
Deep endometriosis	67 (79.7%)	73 (80.2%)	1
Pain intensity (NRS)			
Dysmenorrhea	7 ± 2.8	5.2 ± 3.2	<0.001
NCPP	5.3 ± 3.1	2.4 ± 2.7	<0.001
Dyspareunia	5.9 ± 3.3	1.5 ± 2.4	<0.001
Severe NCPP (NRS ≥ 7)	36 (42.8%)	12 (13.2%)	<0.001

Values are shown as mean ± standard deviation and percentages unless otherwise stated. Abbreviations: BMI, body mass index; NCPP, non-cyclical chronic pelvic pain; SD, standard deviation; MPP, myofascial pelvic pain; NRS, Numerical Rating Scale; y/n, yes/no.

**Table 2:** Comorbidities and other overlapping symptoms in the two groups analyzed.

	MPP Group (n = 84)	Non-MPP Group (n = 91)	*p*-Value
Fatigue (NRS)	6.5 ± 2.5	4.5 ± 2.9	<0.001
Insomnia	50 (60.9%)	39 (43.8%)	0.032
Osteoarticular pain	33 (41.7%)	17 (19%)	0.002
Generalized muscle pain	50 (61.7%)	34 (38%)	0.003
Headache	53(64%)	40 (44%)	0.014
Intestinal rhythm alterations	73 (86.9%)	63 (69.2%)	0.006
Nausea/vomiting	21 (25%)	8 (8.8%)	0.004
Abdominal bloating	61 (72.6%)	33 (36.2%)	<0.001

Values are given as mean ± standard deviation and percentages unless otherwise stated. Abbreviations: MPP, myofascial pelvic pain; NRS, Numerical Rating Scale.

**Table 3:** Scores of the SF-36, HADS, and CSI questionnaires in the two groups analyzed.

	MPP Group (*n* = 84)	Non-MPP Group (*n* = 91)	*p*-Value
SF questionnaire			
Physical functioning	73.8 ± 22.1	87.5 ± 13.8	<0.001
Role physical	53.2 ± 30.1	72.1 ± 23.3	<0.001
Body pain	37.3 ± 23.9	59.7 ± 26	<0.001
General health	39.1 ± 18.6	53.7 ± 22.9	<0.001
Vitality	47.5 ± 14.3	56.3 ± 12.8	<0.001
Social functioning	53.6 ± 26.5	70.9 ± 25.2	<0.001
Role emotional	70.23 ± 27	78.9 ± 23	0.023
Mental health	50.1 ± 24.6	62.2 ± 17.1	<0.001
HADS			
HADA	9.93 ± 4	8.2 ± 3.7	0.004
HADD	7.1 ± 3.9	4.8 ± 3.2	<0.001
CSI total score	50.8 ± 14.9	37.3 ± 15.9	<0.001
CSI score ≥ 40	67 (79.7%)	38 (41.7%)	<0.001

Values are given as mean ± standard deviation. Abbreviations: MPP, myofascial pelvic pain; SF-36, Short Form 36-item; HADS, Hospital Anxiety and Depression Scale; HADA, Hospital Anxiety Scale; HADD, Hospital Depression Scale; MPP, myofascial pelvic pain; CSI, Central Sensitization Inventory; CSI ≥ 40, CSI cutoff score.

**Table 4:** Logistic regression analysis of variables associated with myofascial pelvic pain in patients with endometriosis.

Variable	Odds Ratio	Lower 95% CI	Upper 95% CI	*p*-Value
Severe NCPP (NRS ≥ 7)	2.71	1.20	6.13	0.002
Abdominal bloating	3.33	1.64	6.74	0.004
CSI score ≥ 40	2.76	1.33	5.73	0.009

CI, Confidence interval; NCPP, non-cyclical chronic pelvic pain; NRS, Numerical Rating Scale; CSI, Central Sensitization Inventory; CSI ≥ 40, CSI cutoff score.

## Data Availability

The data presented in this study are available on request from the corresponding author.

## Abbreviations

The following abbreviations are used in this manuscript:

MPP	Myofascial pelvic pain;
HRQoL	Health-related quality of life;
TrPs	Trigger points;
CS	Central sensitization;
CSI	Central Sensitization Inventory;
TVUS	Transvaginal ultrasound;
NRS	Numerical Rating Scale;
NCPP	Non-cyclical chronic pelvic pain;
HADS	Hospital Anxiety and Depression Scale;
SF-36	Short Form 36-item.
